# DEVELOPING AN INTERPROFESSIONAL GRADUATE CERTIFICATE IN LONG-TERM SERVICES AND SUPPORTS

**DOI:** 10.1093/geroni/igac059.317

**Published:** 2022-12-20

**Authors:** Gail Towsley, Leigh Elrod, Nicholas Cox, Alberto Varela, Linda Edelman

**Affiliations:** University of Utah, Salt Lake City, Utah, United States; University of Utah Physician Assistant Studies, Salt Lake City, Utah, United States; University of Utah College of Pharmacy, Salt Lake City, Utah, United States; University of Utah School of Dentistry, Salt Lake City, Utah, United States; University of Utah College of Nursing, Salt Lake City, Utah, United States

## Abstract

The demand for long-term services and supports (LTSS) is increasing and the capacity and competencies of the interprofessional LTSS workforce need to be expanded to provide value-based, person-centered care. The purpose of this presentation is to describe the process of developing an interprofessional LTSS graduate certificate in Gerontology. We broadened an existing nursing graduate certificate to encompass content related to skilled nursing facilities, assisted living and home health and hospice agencies. We will discuss: 1) how we engaged faculty from seven graduate programs: Pharmacy, Physical Therapy, Occupational Therapy, Dentistry, Social Work, Nursing and Physician Assistant to co-create the certificate; 2) challenges and benefits to developing a certificate; and 3) the 15-credit hour certificate components. Our faculty partners were committed to offering a LTSS focused graduate certificate which includes didactic courses and clinical hours. Four students have enrolled in the LTSS graduate certificate in Gerontology since implementation in Spring 2021.

